# Influence of an 8-Week Exercise Program on Physical, Emotional, and Mental Health in Saudi Adolescents: A Pilot Study

**DOI:** 10.3390/medicina59050883

**Published:** 2023-05-04

**Authors:** Afnan Gmmash, Asma Alonazi, Muataz Almaddah, Afnan Alkhateeb, Ohud Sabir, Samiah Alqabbani

**Affiliations:** 1 Department of Physical Therapy, College of Medical Rehabilitation Sciences, King Abdulaziz University, Jeddah 21589, Saudi Arabia; 2 Department of Physical Therapy and Health Rehabilitation, College of Applied Medical Sciences, Majmaah University, Riyadh 11952, Saudi Arabia; 3 Department of Rehabilitation Sciences, College of Health and Rehabilitation Sciences, Princess Nourah bint Abdulrahman University, Riyadh 84428, Saudi Arabia

**Keywords:** physical activity, adolescents, motivation, supervised exercises, mental health

## Abstract

*Background and Objectives:* Physical activity is essential for adolescents to maintain a healthy lifestyle. The aim of this study was to assess the influence of an 8-week exercise program and motivation on physical activity levels, self-motivation, and mental well-being of adolescents in Saudi Arabia. Moreover, the impact of virtual coaching on physical, emotional, and mental health after an eight-week exercise program was examined. *Materials and Methods*: Twenty-seven participants, 18 females (67%) and 9 males (33%), with a mean age of 14 ± 2.38 years, were enrolled in eight weeks of pre- and post-intervention between June and August of 2021. The physical activity scale, situational motivation scale, mental health continuum short form, and baseline assessments were completed before and after the eight-week program. The program recommended that adolescents practice aerobic, resistance, and weight-bearing exercises for 60 min daily. Paired mean t-tests were used to compare pre-and post-test results. *Results:* Participants showed an acceptable physical activity level (5.5 ± 1.4) on a 10-point scale with a significant improvement after the eight-week program (7 ± 1.5; *p* = 0.013). The situational motivation scale improved from 38.1 ± 16 to 26 ± 19.6 (*p* = 0.042). The mental health continuum (social and psychological well-being) also significantly improved. Participants who received weekly phone calls showed similar improvement patterns but were not significantly different from those who did not receive calls. *Conclusions:* A virtually delivered 8-week exercise program for adolescents improved their physical, motivational, and mental health. Providing additional weekly phone calls does not provide additional improvement. Providing adolescents with the needed supervision and motivation enhances their physical activity and mental health.

## 1. Introduction

Routinely practiced physical activity is an integral part of a healthy lifestyle for children of all ages [1]. Children and adolescents are encouraged to participate in physical activity programs throughout the year and especially in the summer when there is a noticeable decline in physical activity [2]. During summer vacation, adolescents’ lifestyles become sedentary as they escape hot weather by engaging in indoor activities. Most of these activities are practiced while sitting, such as watching TV and playing video games [2]. As a result, the prevalence of obesity and physical inactivity has increased, especially with the introduction of virtual gaming and distance learning [3]. Obesity and sedentary behaviors can lead to several chronic disorders that affect several systems such as the cardiovascular, gastrointestinal, and pulmonary systems [4].

Physical activity may promote mental health in children and adolescents, as they develop and grow rapidly. A systematic review and meta-analysis suggested that participating in physical activity with the recommended guidelines can improve overall mental health in children and adolescents, as it may improve anxiety and depression by promoting brain function and elevating dopamine and serotonin levels [3]. Promoting physical activity is essential to prevent mental health problems, such as depression and anxiety, as research suggests that around 20% of adolescents before the age of 18 year experience stress-related problems that affect their mental health [4]. The COVID-19 pandemic exacerbated these behaviors and increased dependency on sedentary leisure activities. Online learning, precautionary measures, lockdowns, and suspension of sports activities have increased the worldwide prevalence of inactivity [3].

The latest recommendations in the second edition of the Physical Activity Guideline for Americans, released by the U.S. Department of Health and Human Services in 2018, stated that adolescents should be engaged in moderate physical activities for at least 60 min daily. Incorporating aerobic, resistance, and weight-bearing exercises are essential to promote cardiovascular health, muscle strength, and bone growth. In addition, regular moderate to vigorous physical activities for adolescents is crucial to promote their physical and mental health [5]. Despite the recommendations and significant health benefits of physical activity, the rate of inactivity is high in adolescents worldwide. In Saudi Arabia, a recent systematic review revealed that most adolescents are inactive, and there is an increasing need for public health programs to reduce sedentary behaviors [6]. Encouraging adolescents to become physically active can be challenging. The provision of extrinsic motivators delivered through peer and parent support is linked with enhanced levels of physical activity [7].

Adolescents are usually motivated to participate in competitive sports activities rather than performing daily physical activities on their own [8]. However, adolescents lack internal motivation, and they often require external compensation that encourages them to practice [9]. Having the intrinsic motivation to be physically active is an essential skill that adolescents should acquire to become physically active constantly throughout their lifespan [10]. Numerous techniques have been created to promote physical activity for adolescents, such as wearable activity trackers and digital resources. However, these strategies were only temporarily effective in increasing adolescents’ physical activity levels [11]. High self-efficacy levels have been linked with successful application of activity programs [12]. Strategies targeting internal motivation have been shown to increase the physical activity of older individuals and obese adolescents. However, more evidence is needed to assist healthy adolescents in developing the intrinsic motivation and self-efficacy using cost effective methods to perform daily exercises during sedentary times such as summer for their well-being [13].

Daily reminders, motivational sessions, and receiving guided phone calls can increase self-motivation and physical activity levels [14,15]. Weekly phone calls from healthcare professionals, such as physical therapists or personal trainers, can be beneficial in encouraging adolescents to overcome environmental and interpersonal barriers that affect their engagement [14]. On-site coaching sessions require constant financial support to cover trainers’ expenses. Alternatively, online-based coaching can be used to promote physical activity levels. Online-based educational studies have been used in the past to assess the effectiveness of interventions in the natural environment and lead to favorable effects [16]. Favorable effects of structured exercises on patient outcomes have been documented in children with different disorders. Improvements in cardiovascular and muscular functions were found following structured exercises in older adults. However, more studies are needed to understand the effect of a cost-effective educational program that focuses on motivating healthy adolescents to meet the daily recommended amount of physical activity. It would also be beneficial to document the effect of applying motivating strategies to enable healthy adolescents to structure their own physical activity program on improving their health outcomes and their overall activity levels [17,18,19,20]. Therefore, the main aim of this study was to assess the impact of virtual 8-week exercise program on improving physical, emotional, and mental health of adolescents. In addition, the influence of self-motivational sessions through weekly phone calls to the study parameters were investigated. Finally, we explored the current physical activity levels, self-motivation, and mental well-being of adolescents in Saudi Arabia during summer breaks. The findings can assist in providing preliminary data on the feasibility of providing adolescents with a brief educational physical activity program and the efficacy of using online coaching with this age group.

## 2. Materials and Methods

### 2.1. Study Design

Pre- and post-experimental study to examine feasibility of the intervention [21].

### 2.2. Participants

**Figure 1:**
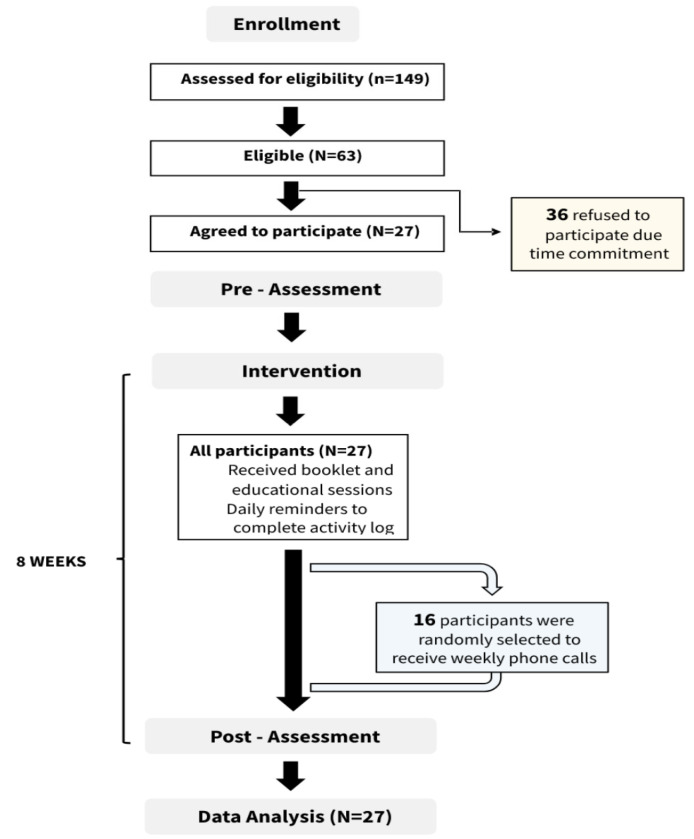
Flow chart of the inclusion process and study protocol.

Participants were recruited through WhatsApp groups, relatives, acquaintances, work colleagues, schools, and pediatric physical therapists throughout Saudi Arabia. A Google form was created and sent to participants interested in participating in the study to assess their eligibility. The inclusion criteria were as follows: aged 10–18 years, being able to write and read in English, and not having any chronic medical condition (orthopedic abnormalities, previous orthopedic surgeries, fractures, mental disorders, cardiopulmonary conditions, significant musculoskeletal injuries, type 1 or type 2 diabetes mellitus, uncorrected visual problems, or other health issues that would interfere with their safety during exercise). From the 149 potential participants, 63 were eligible to participate. Thirty-six withdrew from the study. A flowchart of the inclusion and exclusion criteria and study protocol is shown in Figure 1. The study was conducted according to the guidelines of the Declaration of Helsinki and approved by an appropriate Institutional Review Board on 25 March 2021.

### 2.3. Study Protocol

An eight-week pre-post-intervention study design was used after participants were recruited using convenience sampling. Baseline assessments were completed before starting the eight-week exercise program and after program completion. The participants were randomly assigned to an interventional group receiving additional weekly motivational phone calls (*n* = 16) and the control group who did not receive the motivational phone calls (*n* =11). Pre- and post-assessments were conducted by virtual meetings with all participants and their parents before starting the intervention and at the end of the eight-week period. Participants were divided into four groups, in which there was one blinded assessor with each group to explain each assessment tool to all participants and sent them a translated, detailed, and step-by-step guide to complete each assessment tool.

#### 2.3.1. Eight-Week Exercise Educational and Motivational Program

The eight-week program was created by the study researchers and included the following.

#### 2.3.2. Booklet

In the first section, the researchers created a booklet guided by “the Physical Activity Guidelines for Americans, 2nd edition” [5]. The booklet was available in both Arabic and English versions and included the potential benefits of physical activity and recommended duration, intensity, and type of exercises that are appropriate for their age. The program recommended that adolescents practice aerobic, resistance, and weight-bearing exercises for 60 min daily (10 min of warm-up, 40 min of exercises, and finish with 10 min of cool-down). All the participants and their parents attended a virtual meeting via Zoom. At this meeting, the researchers explained and went through all the points in the booklet and answered the participants’ questions.

#### 2.3.3. Motivational Phone Calls

The second part of the program started by randomly selecting 16 participants to receive weekly motivational calls and to check participants’ adherence. The 16 participants received weekly phone calls from four trained physical therapists concurrently at the beginning of each week. Every physical therapist provided weekly phone calls to the same four participants throughout the intervention period. The duration of the phone calls ranged from 10 to 75 min. The duration of the first call for all the participants ranged from 60 to 75 min. Participants had fewer questions and understood the process more clearly as the study progressed, which shortened the duration of the phone calls for every subsequent week. The average duration of the final phone call was 10–15 min, on average.

The motivation protocol during phone calls was guided by self-determination theory as described by Stone et al. (2009) [22]. The motivational phone calls aimed at supporting the participants’ autonomy, which encouraged them to choose, determine, and act based on their choices. The participants were also provided with positive feedback, active listening, acknowledging their perspectives, and inviting them to solve problems to improve their sense of competence. During the weekly phone calls, the assigned physical therapist viewed the activity daily logs and discussed the participants’ application of physical activity and reasons for not meeting the recommended physical activity. The coach assisted the participants in overcoming obstacles or restrictions that could limit them from meeting the recommended amount of exercise daily. The coach also discussed alternative options for performing the exercises to reach the recommended duration of 60 min under different conditions.

#### 2.3.4. Daily Activity Log

Currently, there is no gold standard for documenting daily physical activities. Activity logs are the most commonly used method by researchers to document the type and amount of physical activity. This was collected via Google Forms and included the type, intensity, and duration of the exercise performed daily. Then, the average amount of minutes exercising per day over the eight weeks was estimated. Each therapist sent two reminders per day in the morning and evening for all participants to complete the activity log.

### 2.4. Outcome Variables

The primary outcome variables for this study were measures of physical activity, self-efficacy, and psychological well-being. At the baseline assessment, participants’ age, sex, height (cm), weight (kg), body mass index (BMI; kg/m^2^), academic level, current physical activity level, and questions regarding their current physical activity participation were documented.

#### 2.4.1. Physical Activity Scale

For the physical activity measure, the self-determination level of activity was used. Participants were asked to rate their activity levels during the day, considering the past seven days. The activities include school education activities, social community activities, and/or spare-time activities. The 10-point scale reflects the activity level, where 10 means active all day and 0 means sedentary all day.

#### 2.4.2. Situational Motivation Scale (SIMS)

This scale was used to measure the participants’ motivational orientation toward their activities. It comprises 16 items measuring intrinsic motivation, identified regulation, external regulation, and amotivation. This scale achieved adequate reliability and constructed validity in adolescents [18]. The participants had to respond to the 16 items per the following scale: 1 = “corresponds not at all” to 7 = “corresponds exactly”. Using the following equation, we calculated the self-determination index. Higher scores indicated higher self-determination.

SDI = [(2 × intrinsic motivation) + identified regulation − external regulation − (2 × motivation)].

#### 2.4.3. Self-Efficacy Scale for Children and Young Adolescents (SEQ-C)

The SEQ-C measures three domains of children’s self-efficacy in affective disorders: social self-efficacy, academic self-efficacy, and emotional self-efficacy. In this study, we only used emotional self-efficacy. Emotional self-efficacy has eight questions pertaining to the perceived capability to cope with negative emotions. The lower the SEQ-C score, the higher the level of depression [23].

#### 2.4.4. Mental Health Continuum Short Form (MHC-SF)

The MHC-SF is derived from the long form MHC, which comprises seven items measuring emotional well-being. For this study, we used the adolescent version, which consisted of 14 statements asking about the frequency of that feeling during the past month. The first three items relate to emotional well-being, items 4–8 measure social well-being, and the final six items measure psychological well-being. A lower score indicates better mental well-being, and a higher score indicates worse mental well-being [24].

#### 2.4.5. Godin Leisure-Time Exercise Questionnaire

GODIN measures the number of times on average the person performs certain types of exercise for more than 15 min (mild, moderate, and strenuous). To find the GODIN Total score, we used the following formula using the times per week for every intensity level. GODIN total score = [(9 × Strenuous) + (5 × Moderate) + (3 × Light)]. Based on the GODIN total score, the physical activity level is determined. The score of 13 or less is interpreted as Sedentary. The score of 14–23 is considered Moderately Active. The score of 24 or higher indicates an Active person [25].

### 2.5. Data Analysis

Data were analyzed using SPSS 21.0 (IBM Corp.: Armonk, NY, USA). Descriptive statistics, including means and standard deviations, were calculated for all demographic and outcome variables. The Shapiro–Wilk test was used to verify the normality of distribution and significance of the obtained results. Paired mean t-tests were conducted to compare pre- and post-test responses for the participants’ variables and outcome measures. Moreover, the Wilcoxon signed-rank test was used to compare the difference in the total Godin, SIMS, and MHC-SF scores within the phone call and no phone calls groups. While the Mann–Whitney test was used to compare between the treatment and control groups in the total of Godin, SIMS, and MHC-SF scores. Significance was set at <0.05.

## 3. Results

### 3.1. Demographic Data

Twenty-seven participants participated in this study. The demographic characteristics of the participants are presented in Table 1.

To investigate our second aim, the results of all participants were combined before and after the eight-week program. A significant change was found in participants’ self-rated scale of daily physical activity level. A positive improvement in participants’ social mental health was also apparent. In addition, psychological well-being improvement was noted following the eight-week program (Table 2). Regarding the third aim, the subgroup that received weekly phone calls was investigated. A similar pattern that was observed in the analysis of 27 participants was also observed in the subgroup results. The documented improvement was on the self-rated physical activity and self-motivation scales of mental well-being (Table 2).

#### 3.1.1. Physical Activity Level

The Wilcoxon signed-rank test revealed no significant difference in the total Godin score within the phone call and no phone calls groups. Moreover, no significant difference was found between the study groups concerning total Godin scores (Table 3). Further, the Mann–Whitney test indicated no significant difference in Godin strenuous, Godin moderate, or Godin light activities. Additionally, no significant differences were found between the groups in the Godin leisure-time exercise questionnaire (Table 4).

#### 3.1.2. Situational Motivation Scale

The Wilcoxon signed-rank test revealed a significant difference within the phone call group in the total SIMS score. However, there was no significant difference in intrinsic motivation, identified motivation, external regulation, or motivation within the group. Additionally, there was no significant difference in the total SIMS score or within the situational motivation scale subdomain. The Mann–Whitney test indicated no significant difference between the phone calls and no phone calls groups in the total SIMS score. Furthermore, no significant differences were observed between the study groups in the situational motivation scale subdomains (Table 5).

#### 3.1.3. Mental Health Continuum SF

Table 6 reveals the results of the mental health continuum between and within the study groups. The Wilcoxon signed-rank test revealed a significant difference in the total MHC-SF score within the phone call group. However, there were no significant differences in the emotional, psychological, or social well-being subscales. Moreover, there was no significant difference in the total MHC-SF score or in the emotional, psychological, and social well-being subscales. The Mann–Whitney test indicated no significant difference between the treatment and control groups in the total MHC-SF score. No significant differences were observed between the study groups in the emotional, psychological, or social well-being subscales.

#### 3.1.4. Daily Activity Log

**Figure 2:**
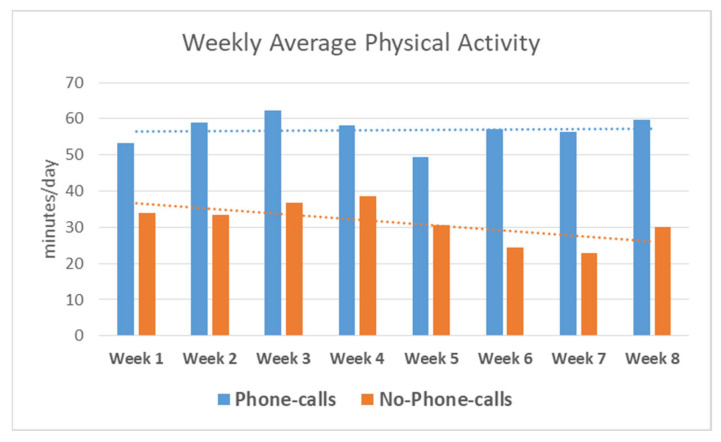
Weekly average physical activity per day in minutes between the phone calls and no phone calls group.

Looking at the daily log of the two groups, we found a significantly higher average of exercises’ minutes per day in the group that received weekly calls than in those who did not receive weekly calls. The average physical activity level per day in minutes across the eight-week protocol is presented in Table 7 and Figure 2.

## 4. Discussion

This study was conducted in Saudi Arabia during the COVID-19 pandemic to provide feasibility and effectiveness of a virtual physical educational and motivational program on physical activity, motivation, and mental wellbeing. The results are encouraging as improvements in physical and mental outcome measures were noted. In addition, participants showed a substantial commitment to documenting their daily activities. However, several limitations restrict the generalizability of the results.

It has been documented that the level of physical activity among adolescents worldwide does not meet minimum requirements [26]. A previous study conducted by Al-Sobayel et al., (2015) showed that adolescents spend approximately 24 min per day engaging in leisure and non-leisure activities, which is below the recommended daily activity for this age group [27]. Although the time spent on daily activities varied among the participants in our study, the participants had an acceptable physical activity level as they were categorized as active using the Godin activity scale. The average BMI was within the normal level (21.96 ± 4.94). These findings may indicate an increase in the physical activity of adolescents in the Saudi community during the summer. Recent governmental efforts and healthy lifestyle campaigns may have affected these results. The Saudi Ministry of Health has been creating campaigns to raise awareness to prevent chronic diseases and promote a healthy lifestyle [28].

This study showed a significant improvement in perceived physical activity levels following the educational program compared with the baseline level. The eight-week protocol enhanced participants’ physical activity level, self-determination index, and social and psychological well-being. These findings support those of a review conducted and suggest that regular physical activity can support children’s and adolescents’ mental health [3]. Enhancing adolescents’ awareness of the effects of physical activity shows a significant improvement in their social and psychological well-being following the physical activity educational program. Although the values of all other variables improved in comparison with the baseline levels, this difference was not significant.

Although no significant difference was found between the two groups in physical and mental health, the amount of time spent exercising differed between the two groups. Participants who received phone calls spent significantly more time exercising than those who did not receive phone calls. The difference between the average daily activities increased with time. These findings can suggest possible benefits from supervision in achieving the recommended physical activity levels, which were not achieved in the other group. A previous systematic review that investigated the prevalence of physical inactivity in Saudi Arabia found that the recommended physical activity level was not met for participants of all ages [6]. Our findings suggest that providing adolescents with individualized motivational calls can increase their ability to participate in physical activity for longer periods daily. The lack of significant difference between groups in all other measures may be owing to the small sample size, as actual improvements were noticed in the individual values; however, they were not significant.

### Limitations

Several limitations may have affected the external validity of our results. This study was underpowered owing to its small sample size. In addition, most participants were girls with high physical activity levels and a normal BMI, which could have influenced the results. The participants were minors, and their parents completed some forms that could have affected the reliability of the entered data. This was an online study, and all measures and assessments were collected virtually. Further studies can benefit from this study and include a larger sample size to better assess the influence on emotional and mental health. Moreover, pre- and post-objective measures collected in person are necessary and should be included in future studies to detect true intervention effects.

## 5. Conclusions

Significant improvements were detected after the provision of the virtual eight-week program. Preliminary data suggest that providing a virtual physical educational program can improve adolescents’ physical and mental health. Providing adolescents with one detailed educational session, a detailed yet simple booklet, and occasional motivational phone calls to assist them in problem-solving can enhance their ability to meet the required amount of physical activity. The addition of supplementary motivational phone calls increased the time spent exercising. Future studies should focus on larger groups of adolescents. The use of a wearable activity tracker is also recommended to document adolescents’ physical activity with and without motivational calls during the summer break.

## Figures and Tables

**Table 1:** Participants’ demographics (*n* = 27).

Demographics	Mean ± SD or *n* (%)	Shapiro–Wilk
Age (years)	14 ± 2.38	0.225
Height (cm)	157.5 ± 11.12	0.552
Weight (kg)	55 ± 15	0.523
BMI (kg/m^2^)	21.96 ± 4.94	0.605
Wake up (hh:mm)	13:00 ± 3:00	0.045
Bedtime (hh:mm)	07:00 ± 08:00	0.005
Total sleeping hours	10 ± 1.5	0.306
Sex		
Female	18 (67%)	
Male	9 (33%)	
City		
Riyadh	12 (44.4%)	
Jeddah	11 (40.8%)	
Yanbou	3 (11.1%)	
Makkah	1 (3.7%)	

**Table 2:** Participants outcome measures pre and post the eight-week program for all participants (*n* = 27), and for the participants who received phone calls (*n* = 16).

Outcome Measure	Pre-Program Mean ± SD	Post-Program Mean ± SD	Paired *t*-Test (*p*)	Pre-Program Mean ± SD	Post-Program Mean ± SD	Paired *t*-Test (*p*)
	*n* = 27	*n* = 27	*n* = 27	*n* = 16	*n* = 16	*n* = 16
Weight (kg)	58 ± 14.5	57 ± 13.4	0.362	55 ± 15	54 ± 13	0.253
Activity scale	5.5 ± 1.4	7 ± 1.5	0.013	5.5 ± 1.4	7 ± 1.5	0.003
Body mass index	22.8 ± 4.7	22.3 ± 4.3	0.203	22 ± 5	21.4 ± 4.3	0.094
SIMS intrinsic motivation	22.5 ± 7	19 ± 6	0.110	23 ± 5.8	19.4 ± 6	0.022
SIMS identified regulation	22 ± 5.7	19.7 ± 6.9	0.314	22.5 ± 5	20.5 ± 7	0.283
SIMS external regulation	17 ± 5.3	15 ± 6	0.258	16.2 ± 5.6	14.4 ± 6.7	0.143
IMS amotivation	6 ± 2.4	8.5 ± 4.7	0.066	6.5 ± 3.6	9.2 ± 5.8	0.054
Self determination Index	38.1 ± 16	26 ± 19.6	0.042	38.6 ± 16	26.5 ± 19.5	0.013
Emotional SEQ	23.5 ± 5	24.4 ± 5.9	0.549	23.5 ± 4.8	24.5 ± 5.7	0.390
MHS emotional well-being total	9.7 ± 2.6	11.1 ± 4.1	0.134	9.5 ± 2.8	11.3 ± 3.3	0.011
MHS social well-being total	12.9 ± 4.8	16.6 ± 6.5	0.021	12.9 ± 5	16 ± 5.8	0.009
MHS psychological well-being	17 ± 7.3	20.6 ± 8.2	0.045	18.3 ± 6.3	20.5 ± 7.3	0.075

SIMS: situational motivation scale; SEQ: self-efficacy questionnaire; MHS: mental health score.

**Table 3:** Comparison between the two studied groups according to the level of Godin leisure-time exercise questionnaire.

Godin Leisure-Time Exercise Questionnaire	Phone Calls Group		No Phone Calls Group		
	Pre (*n* = 16)	Post (*n* = 16)	Pre (*n* = 11)	Post (*n* = 11)	Test of Sig. (*p*)
	*n* (%)	*n* (%)	*n* (%)	*n* (%)	
Active	13 (81.3%)	13 (81.3%)	7 (63.6%)	9 (81.8%)	c^2^ = 0.529 (^MC^*p* = 1.0)
Moderately active	1 (6.3%)	2 (12.5%)	3 (27.3%)	1 (9.1%)	
Not active	2 (12.5%)	1 (6.3%)	1 (9.1%)	1 (9.1%)	
MH (*p*_0_)	11 (0.637)		11 (0.346)		
Total Godin Leisure-time					
Mean ± SD	38.25 ± 22.81	47.88 ± 25.92	33.55 ± 26.28	44.55 ± 24.20	U = 79.5 (0.680)
Median	36.5	42.5	28	40	
Z (*p*_0_)	1.216 (0.224)		1.067 (0.286)		

Note. SD: standard deviation; U: Mann–Whitney test; Z: Wilcoxon signed-rank test; c^2^: chi-square test; ^MC^: Monte Carlo; MH: marginal homogeneity test; *p*_0_: *p*-value for comparing between pre and post in each group; *p*: *p*-value for comparing between the studied groups.

**Table 4:** Comparison between the two studied groups according to the level of Godin leisure-time exercise questionnaire.

Godin Leisure-Time Exercise Questionnaire	Phone Calls Group (*n* = 16)		No Phone Calls Group (*n* = 11)		Test of Sig. (*p*)
	Pre	Post	Pre	Post	
	M ± SD	M ± SD	M ± SD	M ± SD	
Strenuous	1.88 ± 1.26	2.81 ± 1.94	1.73 ± 1.79	2.55 ± 2.21	U = 75.0 (0.544)
Z (p_0_)	1.404 (0.160)		0.672 (0.502)		
Moderate	3.0 ± 1.83	2.94 ± 1.61	2.18 ± 1.66	2.91 ± 1.92	U = 82.500 (0.790)
Z (p_0_)	0.040 (0.968)		0.976 (0.329)		
Light	2.13 ± 2.33	2.63 ± 2.16	2.36 ± 1.80	2.36 ± 2.38	U = 74.500 (0.512)
Z (*p*_0_)	0.866 (0.386)		0.213 (0.832)		
Total Godin Leisure-time	38.25 ± 22.81	47.88 ± 25.92	33.55 ± 26.28	44.55 ± 24.20	U = 79.500 (0.680)
Z (*p*_0_)	1.216 (0.224)		1.067 (0.286)		

Note. SD: standard deviation; U: Mann–Whitney test; Z: Wilcoxon signed-rank test; *p*_0_: *p*-value for comparing between pre and post in each group; *p*: *p*-value for comparing between the studied groups.

**Table 5:** Comparison between the two studied groups according to the situational motivation scale.

Situational Motivation Scale	Phone Calls Group (*n* = 16)		No Phone Calls Group (*n* = 11)		U (*p*)
	Pre M ± SD	Post M ± SD	Pre M ± SD	Post M ± SD	
Intrinsic motivation	22.56 ± 7.04	19.19 ± 6.21	22.91 ± 3.83	19.73 ± 6.20	84 (0.865)
Z (*p*_0_)	1.838 (0.066)		1.684 (0.092)		
Identified regulation	22.0 ± 5.69	19.75 ± 6.94	23.18 ± 4.53	22.0 ± 6.32	68.5 (0.342)
Z (*p*_0_)	1.162 (0.245)		0.409 (0.683)		
External regulation	17.0 ± 5.30	15.06 1± 6.06	14.91 ± 6.04	13.45 ± 7.93	74 (0.512)
Z (*p*_0_)	1.166 (0.244)		1.186 (0.236)		
Amotivation	6.0 ± 2.45	8.50 ±1 4.76	7.36 ± 4.97	10.36 ± 7.23	69 (0.368)
Z (*p*_0_)	1.694 (0.090)		1.130 (0.258)		
SDI	38.13 ± 16.03	26.06 ± 19.68	39.36 ± 16.88	27.27 ± 20.66	84.5 (0.865)
Z (*p*_0_)	1.967 (0.049)		1.468 (0.142)		

Note. SD: standard deviation; U: Mann–Whitney test; Z: Wilcoxon signed-rank test *p*_0_: *p*-value for comparing between pre and post in each group; *p*: *p*-value for comparing between the studied groups.

**Table 6:** Comparison between the two studied groups according to the adolescent mental health continuum (MHC-SF).

Adolescent Mental Health Continuum	Phone Calls Group (*n* = 16)		No Phone Calls Group (*n* = 11)		*t* (*p*)
	Pre M ± SD	Post M ± SD	Pre M ± SD	Post M ± SD	
Emotional	9.69 ± 2.65	11.13 ± 4.10	9.27 ± 3.13	11.45 ± 1.75	0.286 (0.778)
*t*_1_ (*p*_0_)	1.583 (0.134)		2.564 (0.028)		
Psychological	17.06 ± 7.34	20.63 ± 8.20	20.09 ± 4.25	20.27 ± 6.20	0.120 (0.905)
*t*_1_ (*p*_0_)	2.192 (0.045)		0.117 (0.909)		
Social well-being	12.94 ± 4.77	16.63 ± 6.48	12.82 ± 5.51	15.09 ± 4.83	0.667 (0.511)
*t*_1_ (*p*_0_)	2.577 (0.021)		1.289 (0.227)		
Total MHC-SF	39.69 ± 13.84	48.38 ± 18.06	42.18 ± 10.10	46.82 ± 11.86	0.250 (0.804)
*t*_1_ (*p*_0_)	2.482 (0.025)		1.353 (0.206)		

Note. SD: standard deviation; *t*: student’s *t*-test; *t*_1_: paired *t*-test; *p*_0_: *p*-value for comparing between pre and post in each group; *p*: *p*-value for comparing between the studied groups.

**Table 7:** Average physical activity level per day in minutes across the eight-week protocol.

Week	Phone Calls Group (*n* = 16)	No Phone Calls Group (*n* = 11)		
	Average Physical Activity min/day	Average Physical Activity min/day		
1	53.24 ± 17	34.00 ± 20		
2	58.96 ± 16.5	33.33 ± 19		
3	62.36 ± 8.8	36.75 ± 21		
4	58.19 ± 12.3	38.67 ± 26		
5	49.51 ± 21	30.67 ± 25		
6	57.22 ± 10	24.33 ± 26		
7	56.42 ± 21	22.92 ± 21		
8	59.79 ± 13	30.17 ± 28		
Physical activity min/day (Average over eight weeks)	56.96 ± 3.75	31.35 ± 5.2	*p* < 0.001	

## Data Availability

Data sharing is unavailable due to privacy.
